# Possible Role of Interleukin-1β in Type 2 Diabetes Onset and Implications for Anti-inflammatory Therapy Strategies

**DOI:** 10.1371/journal.pcbi.1003798

**Published:** 2014-08-28

**Authors:** Gang Zhao, Gitanjali Dharmadhikari, Kathrin Maedler, Michael Meyer-Hermann

**Affiliations:** 1 Department of Systems Immunology and Braunschweig Integrated Centre of Systems Biology, Helmholtz Centre for Infection Research, Braunschweig, Germany; 2 Centre for Biomolecular Interactions Bremen, University of Bremen, Bremen, Germany; 3 Institute of Biochemistry, Biotechnology and Bioinformatics, Technische Universität Braunschweig, Braunschweig, Germany; National Institutes of Health, United States of America

## Abstract

Increasing evidence of a role of chronic inflammation in type 2 diabetes progression has led to the development of therapies targeting the immune system. We develop a model of interleukin-1β dynamics in order to explain principles of disease onset. The parameters in the model are derived from *in vitro* experiments and patient data. In the framework of this model, an IL-1β switch is sufficient and necessary to account for type 2 diabetes onset. The model suggests that treatments targeting glucose bear the potential of stopping progression from pre-diabetes to overt type 2 diabetes. However, once in overt type 2 diabetes, these treatments have to be complemented by adjuvant anti-inflammatory therapies in order to stop or decelerate disease progression. Moreover, the model suggests that while glucose-lowering therapy needs to be continued all the way, dose and duration of the anti-inflammatory therapy needs to be specifically controlled. The model proposes a framework for the discussion of clinical trial outcomes.

## Author Summary

Insulin resistance and relative insulin deficiency are two hallmarks of type 2 diabetes. While insulin resistance is always present in the early phase, it is β-cell failure that determines the pace of the disease onset. Increasing evidence that the immune system is activated and plays an important role in type 2 diabetes has stimulated efforts of developing drugs targeting inflammatory cytokines. We built a model to describe the principles of type 2 diabetes onset under the influence of interleukin-1β. The disease onset is understood in terms of bifurcation. It is found that inflammatory cytokines are required to be suppressed for a limited time only, while glucose has to be controlled over the long term. These structural insights may serve as a guideline for future clinical trials.

## Introduction

Despite more than 350 million patients worldwide and the concomitant expensive socioeconomic burden, the pathogenesis of type 2 diabetes (T2D) is not yet completely understood. T2D is a progressive disease. The most important physiological components of T2D are insulin resistance, which is characterized by impaired response to insulin in insulin-sensitive tissues, and β-cell failure, which is characterized by β-cell dysfunction and reduced β-cell mass. The progression of T2D is clearly divided into at least two phases, pre-diabetes and overt diabetes [1]–[5]. In the pre-diabetes phase, insulin resistance is compensated by increased single β-cell secretion capacity and/or β-cell number. If insulin resistance is not completely compensated, the blood glucose level would grow slowly, manifested as higher fasting glucose (impaired fasting glucose, IFG) and/or higher post-load glucose (impaired glucose tolerance, IGT) [6]. Overt T2D is characterized by compensation failure and continuous loss of functional β-cells [7], [8], hence accompanied by continuously aggravated hyperglycaemia.

Although insulin resistance is usually present in the early phase of pre-diabetes, it is the pace of β-cell failure that determines the onset of overt T2D [9]. The mechanisms leading to the transition from pre-diabetes to overt T2D are unclear [10]. However, there is evidence that the transition from β-cell compensation to β-cell failure happens in a comparably short time span [2], [4], typically within 3 years [2]. This is further supported by a recent longitudinal study in a large population [5]. The trajectory of glycaemia before diagnosis of T2D was shown to be composed of a slow and stable adaptation, which lasts 12 years, followed by a rapid rise of glucose to overt T2D within 2 years [5]. Once overt T2D is started, hyperglycaemia continues to worsen regardless of treatments based on oral anti-diabetic agents [11]–[13].

The evidence of a role of inflammatory responses in the pathogenesis of T2D was increasing in recent years. Interleukin-1β (IL-1β) has been reported to contribute to β-cell failure [14]–[18]. β-cells themselves secrete IL-1β upon glucose stimulation [14]. Furthermore, IL-1β stimulates its own production in β-cells [17] and attracts macrophages [18] which can act as an extra source of IL-1β and other cytokines. Although it is currently unclear whether inflammatory responses are a primary cause or a secondary effect in T2D progression, therapies targeting IL-1β have shown encouraging progress albeit diverse results in different clinical trials [19]–[26].

These results motivated our working hypothesis that the pre-diabetic and overt T2D might be characterised by two qualitatively different states and that IL-1β is a potential candidate for promoting the transition between these two states. The hypothesis is consistent with results from clinic trials in which IL-1β blockade by interleukin-1-receptor antagonist (IL-1Ra), a naturally occurring competitive inhibitor of IL-1β, induced sustained improvements of β-cell function and the systemic inflammation state in patients with a mean disease duration of 11 years, even 39 weeks after cessation of treatment [19], [20]. IL-1Ra competes with IL-1β for IL-1 receptors but does not trigger any signalling event. It is an important regulator of the effect of IL-1β in many cell types, including human pancreatic β-cells which secrete IL-1Ra themselves [27], [28]. In several liver, autoimmune, and infectious diseases IL-1Ra is a better indicator of disease severity than IL-1β [29]. Results from a recent longitudinal study show that an accelerated increase in circulating IL-1Ra starts about 5 years before diagnosis of T2D [30], coinciding with the accelerated deterioration of insulin-sensitivity and the compensation of β-cell function [5]. In addition, long-term effects of temporary intensive insulin therapy on diabetes remission (or at least a prolonged normoglycaemia phase) in newly diagnosed diabetic patients may be partially attributed to the anti-inflammatory activity of insulin [31]. All these results highlight the role of inflammatory responses in the pre-diabetes phase and the possibility that the long-term effects induced by temporary anti-inflammatory therapy are mediated by a switch, i.e. a sudden transition, between the qualitatively different compensation and overt T2D states. Interestingly, *in vitro* experiments show that the effect of IL-1β on β-cell mass and insulin secretion is twofold [32]: low concentration of IL-1β stimulates β-cell proliferation, inhibits β-cell apoptosis and enhances glucose-stimulated insulin secretion while high concentration of IL-1β has opposite effects. In other words, IL-1β may contribute to both the β-cell compensation phase and the β-cell failure phase.

Here, we present a mathematical model showing the possibility that T2D onset is induced by a sudden transition of IL-1β to a high level. The IL-1β switch results from the coexistence of two different states, which is often termed as bi-stability. Bi-stable switch models have been widely used in modeling developmental processes, such as cell cycle progression, cellular differentiation and apoptosis. The characteristics of bi-stable switches include sudden transition, and hysteresis. The latter means that the state of a system depends not only on the current environment but also on its past state. We combine the IL-1β bi-stable switch model with a previously published T2D progression model [33], by assuming extrapolated β-cell turnover rates caused by exogenous IL-1β [32]. Similar concepts describing diabetes progression by different steady states have been already developed for both type 1 and 2 diabetes [33]–[35]. Our model falls into the same class as those previously published by Topp et al. [33] and De Gaetano et al. [35], which considered the evolution of β-cell mass, insulinemia and glycaemia over a time-scale of years and rely on glucose toxicity. β-cell mass is controlled by an empirical parabolic function of glucose in [33]. Subsequently a physiologically amenable concept of pancreatic reserve was introduced, which controls the direction of β-cell mass change [35]. Both β-cell mass and the pancreatic reserve were modeled phenomenologically as functions of glucose, where some parameters bear the potential to act as bifurcation parameters. The model presented here is built on these results. The phenomenological implementation of glucose toxicity is replaced by a model of IL-1β, which is explicitly defined and relatively easy to measure. We focus on an IL-1β bi-stable switch giving rise to hysteresis which is not discussed in previous studies. IL-1β hysteresis turns out to be of utmost importance because it widely shapes the strategy of anti-inflammatory therapies. While the complexity of the disease will never be captured by a mathematical model, the structural insights suggested by the model will help exploiting treatment approaches more efficiently and elaborating the effect of different therapies on β-cell mass and disease progression.

The IL-1β bi-stable switch model implies that T2D onset and later irreversible progression of β-cell failure arise from altered stability properties of the β-cell compensation state. Therefore, the strategy of glucose control would be more effective in the compensation phase than after the onset of overt T2D. This model result is consistent with recent data from the Diabetes Prevention Program Outcome Study (DPPOS) showing that IGT people who once regressed to normoglycaemia have a 56% lower relative risk of developing diabetes [36]. While glucose-lowering therapy bears the potential of stopping disease progression in the compensation phase, the IL-1β bi-stable switch model suggests that a combined glucose-lowering and anti-inflammatory therapy is necessary to stop the loss of functional β-cell mass as well as hyperglycaemia progression in the overt T2D phase, and eventually reestablish the compensation phase.

## Materials and Methods

**Figure 1:**
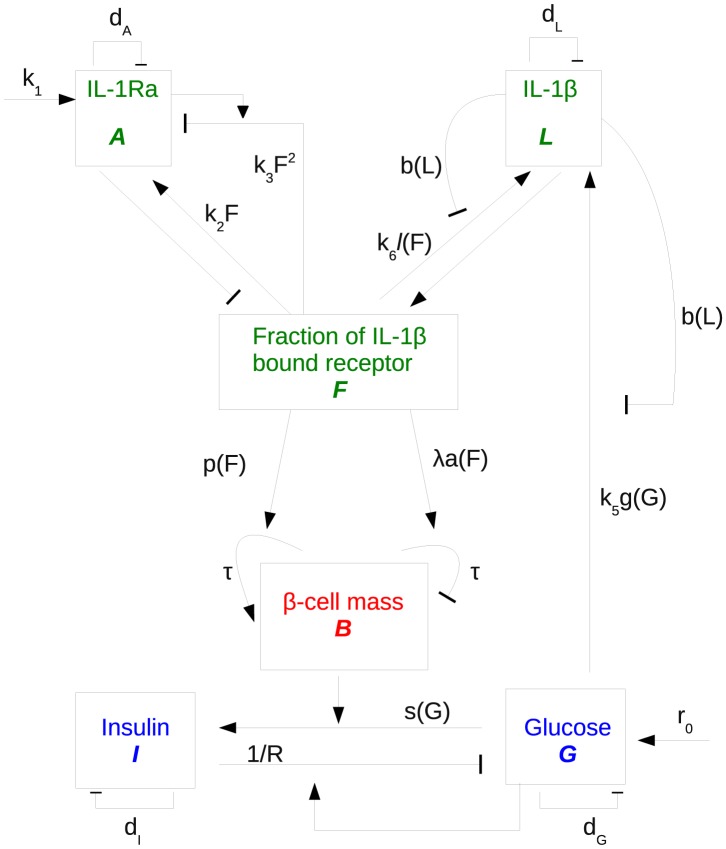
Model scheme: IL-1Ra (*A*) and IL-1β (*L*) compete for IL-1 receptors giving rise to a fraction of IL-1β bound receptors (*F*) which determines subsequent signalling in β-cells (*B*). These control insulin (*I*) release and, via the influence of insulin resistance, blood glucose level (*G*). Arrows: activation effect, line with bar end: inhibition effect. Kinetic terms corresponding to each interaction are labelled. Variables evolving on different time scales are marked by different colours.

A full description of the model (Fig. 1), the assumptions, and the derivation of all model parameters from experimental data (summarized in Table 1) are provided in this section. Scripts of all calculations are provided in the supplement, including the model file and the parameter estimation procedures.

**Table 1:** Summary of parameters used in the model.

Description	Symbol	Value	Unit	Ref.
glucose	*G*			
initial value		5.47( 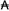 )	mM	[5]
		5.25( 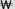 )	mM	
glucose production	*r*_0_	48	mM d^−1^	[33]
insulin-independent Glucose uptake	*d*_G_	1.44	d^−1^	[33]
insulin	*I*			-
initial value		70.73( 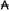 )	pM	DBSS
		74.28( 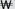 )	pM	
maximal rate of insulin secretion by β-cells	*h*	300.02	pM d^−1^	[33]
insulin clearance rate	*d_I_*	432	d^−1^	[33]
glucose stimulated insulin secretion	*s*(G)			-
	*K*_G_	7.86	mM^2^	[33]
β-cell mass	*B*			-
initial value		312.1( 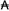 )	mgmg	DBSS
		346.7( 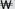 )		DBSS
ratio of proliferation to apoptosis	*λ*	2.33		DBSS
β-cell proliferation	*p*(F)			-
	*a*_p_	−0.048		by fit
	*b*_p_	−0.46		by fit
	*c*_p_	0.62		by fit
β-cell apoptosis rate	*a*(F)			-
	*a*_a_	0.047		by fit
	*b*_a_	0.57		by fit
	*c*_a_	2.36		by fit
β-cell turnover time constant	*τ*	0.00052	d^−1^	FBNH
IL-1Ra	*A*			
initial value		6000( 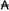 )	pg ml^−1^	*
		5982( 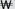 )	pg ml^−1^	DBSS
IL-1β stimulated IL-1Ra production				-
	*k*_1_	9.47e5	*pg* *ml^−1^* *d^−1^*	DBSS
	*k*_2_	3.12e7	*pg* *ml^−1^* *d^−1^*	DBSS
	*k*_3_	2.65e4	*d^−1^*	DBSS
degradation rate of IL-1Ra	*d*_A_	166.36	d^−1^	[29]
dissociation constant of IL-1Ra	*K*_A_	1300	pg ml^−1^	[29]
IL-1β	*L*			
initial value		12( 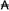 )	pg ml^−1^	*
		11.25( 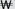 )	pg ml^−1^	DBSS
inhibitory effects of basal IL-1β mRNA	*b*(L)			-
	*u*	5.8		by fit
	*r*	2		by fit
coefficient relating IL-1β mRNA level to its concentration	*k*_4_	0.047	pg^−1^ ml	DBSS
glucose stimulated IL-1β production	*g*(G)			-
	*s*	2.77		by fit
	*v*	3.52	mM	by fit
	*k*_5_	3.745e3	pgml^−1^ d^−1^	DBSS
IL-1β auto-stimulation	*l*(F)			
	*t*	3		by fit
	*K*_F_	0.1575		by fit
	*k*_6_	1.048e9	pgml^−1^ d^−1^	DBSS
degradation rate of IL-1β	*d*_L_	55.45	d^−1^	[63]
dissociation constant of IL-1β	*K*_L_	1300	pg ml^−1^	[29]
insulin resistance	*R*			
	*R*_0_	9.643	pM d	[33]
	*m*	1.688	pM d	FBNH

Parameter values of the mathematical model and references. 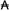: initial value of incident diabetes cases; 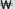: initial value of non-diabetic control; FBNH: fitted by glucose history; DBSS: determined by steady state conditions;

*: parameter discussed in the section “steady state conditions”.

### Overview

The mathematical model (Fig. 1) is constructed in three steps. First, we build the core of the model which describes the competition between IL-1β and IL-1Ra, where glucose stimulates IL-1β production. Since the effect of IL-1β on β-cells is mediated by the binding to its receptor, which is also the target of IL-1Ra antagonism, the fraction of IL-1β bound receptor *F* is used as a measure for IL-1β stimulation.

Second, the interaction between glucose (G) and insulin (I) is modeled with β-cell mass being a parameter. This subsystem is mainly adopted from [33], which is a single-compartment model justified by the slow dynamics of glucose and insulin over a time-scale of days to years. G and I are associated with long-term fasting glucose and insulin levels. As short-term (daily) fluctuations are averaged out, the model cannot describe disease onset derived from details of glucose and insulin dynamics.

Third, the two subsystems which describe the inflammatory signals and glucose-dependent insulin secretion are connected. For that purpose the IL-1β-dependent β-cell turnover rates are adopted from human islets which were cultured with exogenous IL-1β. The data suggest a bimodal effect of IL-1β on the β-cell mass [32]: IL-1β stimulates β-cell proliferation and inhibits β-cell apoptosis when presented in low concentrations. Conversely, at high concentrations IL-1β enhances β-cell apoptosis and reduces β-cell proliferation. The unknown model parameters are determined from steady state conditions of the IL-1β/IL-1Ra subsystem and from the natural history of fasting glucose in the pre-diabetic phase [5].

There are three different time scales in the model (Fig. 1). The IL-1β/IL-1Ra subsystem evolves on the fastest scale, since ligands and receptors bind/unbind on a time scale of seconds and proteins are synthesised/secreted on a time scale half an hour. The glucose/insulin subsystem evolves on an intermediate time scale of days [33]. β-cell evolves on the slowest scale. The turnover rate of β-cell is rather slow: typically, one or two cells divide per month. Since our main interest lies on long-term evolution of fasting glucose, the IL-1β/IL-1Ra subsystem is assumed in steady-state equilibrium and β-cell mass is assumed to be constant on this relatively fast time scale of fasting glucose. The different time-scales in the model make it possible to separate the model into different subsystems and justify our three-step modelling approach.

### The antagonist effect of IL-1Ra (*A*)

All effects of IL-1β (*L*) are mediated by the IL-1 receptor and are blocked by IL-1Ra (*A*). IL-1Ra competes with IL-1β for the receptor but does not trigger any signalling event. Therefore, ligand-receptor binding and unbinding are considered to describe the antagonist effect of IL-1Ra. The ligand-receptor binding/unbinding happens on a time scale of seconds and is fast compared to other time-scales in the model, such as IL-1β and IL-1Ra production and secretion which happens on a time-scale of tens of minutes. Therefore, it is reasonable to assume that ligand and receptor are always in equilibrium, which enables us to describe the fraction of IL-1β bound receptors (*F*) by
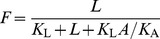
(1)where *K*_L_ and *K*_A_ are the dissociation constants of IL-1β and IL-1Ra, respectively. We assume a constant number of IL-1 receptors on the β-cell membrane. Consequently, *F* can be considered as a measure of IL-1β stimulation.

### Estimate of endogenous IL-1β (*L*) and IL-1Ra (*A*) in cultured islets

*F* is used in the following to fit certain functions in the model to corresponding experimental data. In these experiments, different amounts of exogenous IL-1β were added to cultured human islets and the corresponding effects were measured, while endogenous IL-1Ra and IL-1β were not measured. It is safe to neglect endogenous IL-1β and IL-1Ra when exogenous IL-1β is dominant, which is the case for most measured data points. However, it is necessary to estimate the endogenous IL-1β and IL-1Ra concentrations for data points with zero exogenous IL-1β.

Secretion of IL-1Ra from cultured human islets was measured after 4 days which led to the endogenous IL-1Ra concentration of 114 pg/ml [32]. Endogenous IL-1β is estimated to be 0.228 pg/ml using the ratio of IL-1Ra/IL-1β of 500 [27].

### IL-1Ra (*A*) dynamics

IL-1Ra (*A*) stimulation by IL-1β (*L*) is bimodal [32]. IL-1Ra is induced by IL-1β at exogenous concentrations below 20 pg/ml. At higher concentrations, IL-1β restores IL-1Ra to nearly the basal level. This nonlinear effect is phenomenologically captured by the nonlinear equation
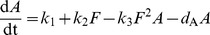
(2)where *d*_A_ is the natural degradation rate of IL-1Ra. The parameters *k*_1_ to *k*_3_ are determined in the Section “steady states conditions” (see below).

### IL-1β (*L*) dynamics

IL-1β dynamics are described by
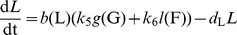
(3)Glucose induces β-cells to secrete IL-1β [17] (*k*_5_ *g*(G)). IL-1β is also secreted from islets via auto-stimulation [17] (*k*_6_ *l*(F)). High levels of IL-1β mRNA inhibit the stimulating effect of glucose and of auto-stimulation [17] (*b*(L)). IL-1β degrades with a rate *d*_L_. In the following, the functions *g*(G), *l*(F), and *b*(L) are derived from experimental data. The parameters *k*_5_ and *k*_6_ are determined in the Section “steady state conditions”.

#### 1. IL-1β induction by glucose (*g*(*G*))

**Figure 2:**
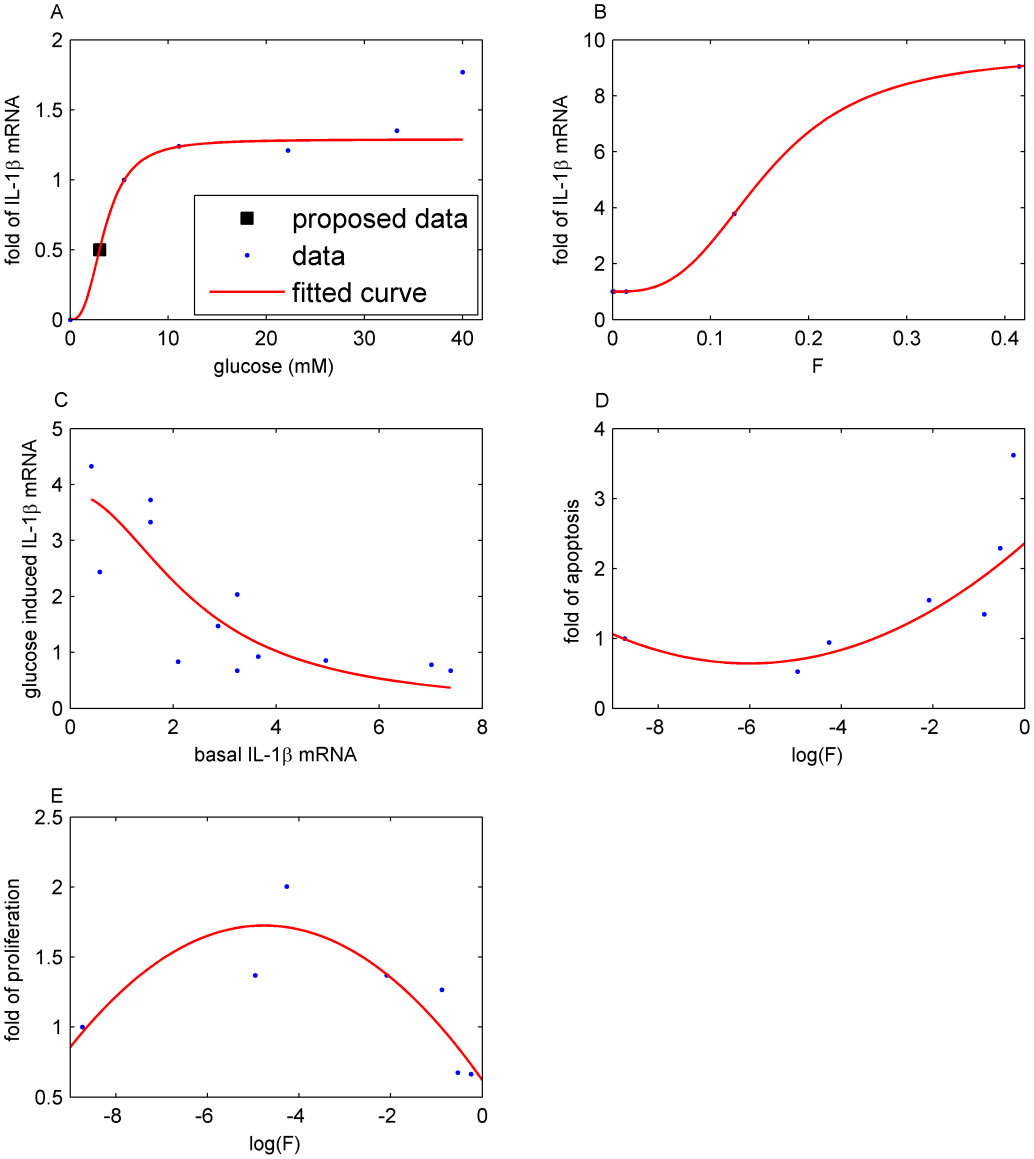
Parameter fit from experimental data. A: Data are reproduced from Fig. 4A in [17]. The function *g(G)* in Eq. (4) is fitted to these data. Note *a* = 1 is used in the model, since the constant could be merged with *k*_5_. B: Data were reproduced from Fig. 4B in [17]. The function *l*(F) in Eq. (5) is fitted to these data. Note *a* = 1 is used in the model. C: Data are reproduced from Fig. 1E in [17]. Eq. (6) is fitted to these data. Note the interchanged axes and *a* = 1 is used in the model. D: Data are reproduced from Fig. 1C in [32]. The function *a*(F) in Eq. (13) is fitted to these data. E: Data are reproduced from Fig. 1B in [32]. The function *p*(F) in Eq. (14) is fitted to these data.

We use a Hill function *g*(*G*)
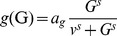
(4)to fit data from cultured human islet describing the increase of IL-1β mRNA (Fig. 4A in [17]) as a function of glucose (Fig. 2 A). This implicitly assumes that IL-1β mRNA level is proportional to IL-1β concentration. Since the available data do not include glucose levels below 5.5 mM, we assume that half of the control rate is achieved at 3 mM (marked in Fig. 2A by the black square), which is the usual limit of hypoglycaemia. The choice of this value does not alter the model results. Other values, such as 2 or 4 mM, only affect the values of the model parameters (*k*_1–6_) determined by steady state conditions (see Section below). We ignore the data point at 40 mM glucose, since such a high glucose level is not physiological. *a_g_* = 1 is used in the model, since the constant is merged with *k_5_*.

#### 2. IL-1β auto-stimulation (*l*(*F*))

IL-1β auto-stimulation is described by
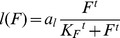
(5)and is fitted to data (Fig. 4B in [17]) which determine the increase of IL-1β mRNA of cultured human islets in dependence on exogenous IL-1β concentrations (Fig. 2 B). *a_l_* = 1 is used in the model, since the constant is merged with *k_6_*.

#### 3. Inhibition of the effects of glucose and IL-1β by IL-1β mRNA (*b*(*L*))

The stimulatory effect of glucose is inhibited by basal IL-1β mRNA [17]. IL-1β auto-stimulation is linearly related to glucose stimulated IL-1β in human islet preparations [17]. This linear relationship suggests a common regulatory mechanism of glucose-stimulated and auto-stimulated IL-1β production. This is represented in Eq. (3) by basal IL-1β mRNA-dependent inhibition of both glucose stimulation and IL-1β auto-stimulation using the sigmoidal function
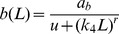
(6)where the scaling parameter *k*_4_ relates IL-1β mRNA level to IL-1β protein concentration. Eq. (6) is fitted to the data (Fig. 1E in [17]) as a function of basal IL-1β (Fig. 2 C) and *k*_4_ is determined in the Section “steady state conditions”. *a_b_* = 1 is used in the model, since the constant is merged with *k_5_* and *k_6_*.

### Glucose (*G*) and insulin (*I*) dynamics

G and I are quantities associated with long-term changes (days to years) of fasting levels of glucose and insulin, respectively. Therefore, a single-compartment model for glucose and insulin is used. The ordinary differential equations describing glucose and insulin are adapted from Topp et al. [33] with the modification that the dynamics of insulin resistance (*R*) is included as input to the model.

#### 1. Glucose dynamics (*G*)

Glucose is produced with a constant rate (*r*_0_) and consumed by an insulin-dependent (*I/R*) and an insulin-independent rate (*d*_G_)
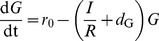
(7)where *R* denotes insulin resistance.

#### 2. Insulin dynamics (*I*)

Insulin dynamics is modeled by a mass balance equation
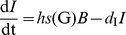
(8)with a secretion rate *s*(G) and a clearance rate (*d*_I_). Secretion is stimulated by glucose according to the sigmoidal function
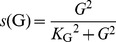
(9)and is proportional to the β-cell mass (*B*). The maximal rate of insulin secretion *h*, the insulin clearance rate *d*_I_, and the half concentration *K_G_* of glucose stimulated insulin secretion are taken from [33].

#### 3. Insulin resistance (*R*)

Insulin sensitivity provided in [5] is homeostatic model assessment (HOMA) index. The one compartment model of glucose and insulin dynamics is a simplification of the minimal model [37]. Insulin resistance derived from the minimal model exhibits a linear relationship to the HOMA-IR index on logarithmic scale [38]. Hence,
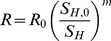
(11)is used to convert the HOMA index value into *R*. Here *S_H_* is the insulin sensitivity index (HOMA2 %S) reported in [5]. The parameter *m* is determined by fitting the model to the natural history of fasting glucose in the pre-diabetic phase [5].

### *β*-cell mass dynamics (*B*)

The dynamics of the β-cell mass
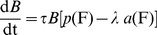
(12)is controlled by IL-1β induced β-cell apoptosis and proliferation. The apoptosis rate *a*(F)
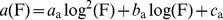
(13)and the proliferation rate *p*(F)
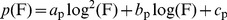
(14)are fitted to data from human islets [32], which were cultured for 4 days with different IL-1β concentrations applied at day 0 (Fig. 2 D and E, respectively).

We assume that the initiation of apoptosis and proliferation is faster than IL-1β-induced IL-1β and IL-1Ra secretion. Thus, the IL-1β and IL-1Ra dynamics are de-coupled from β-cell turnover and the initial *F* (at day 0 of the experiment) can be used as an approximation in fitting the data. Since the apoptosis and the proliferation rates depend on log(F), eventual modifications of *F* caused by newly secreted molecules would anyway remain small on the logarithmic scale.

β-cell apoptosis and proliferation were measured as percentage of control (i.e. no IL-1β added) [32] such that the absolute rates remained undetermined. Scaling of the rates is represented by an extra parameter *τ* (Eq. (12)), which is fitted to the natural history of fasting glucose in the pre-diabetic phase [5]. The relative importance of apoptosis and proliferation is controlled by the parameter *λ* in Eq. (12), which is determined in the Section “steady state conditions”.

### Steady state conditions

While a large number of model parameters could be determined by experimental constraints, the parameters *k*_1–6_ and *λ* remained undetermined and are derived from steady state conditions for Eqs. (2,3,12). Eq. (12) is used to determine *λ*. Eq. (2) and (3) each exhibit two stable steady states and a bifurcation point, such that these 6 additional conditions can be used to determine *k*_1–6_.

#### 1. Steady state of the compensation phase

Reports from the Whitehall II longitudinal study [5] show that 13 years before diagnosis of T2D, the fasting glucose level of the incident diabetic participants is already higher than that of control (5.47 vs. 5.25 mM, Table 2). The corresponding serum IL-1Ra concentration of the incident diabetic participants is 300 pg/ml [30]. Using the reported *in vitro* ratio of IL-1Ra/IL-1β, which is 500 [27], the corresponding serum IL-1β concentration is estimated to be 0.6 pg/ml. This value is consistent with the average serum IL-1β level (0.57±0.93 pg/ml) reported in [39]. Since IL-1β/IL-1Ra are locally produced in the pancreas islets and their half-lives are short (5∼10 minutes), we assume 20 fold more IL-1β/IL-1Ra in the pancreas than in the blood under healthy conditions (note that the blood-tissue ratio might change after T2D onset), inferring 12 and 6000 pg/ml (Table 2) of IL-1β and IL-1Ra, respectively, in the islets. As this choice is arbitrary, other choices of the tissue-blood ratio, such as 200 or 2000, with fixed ratio of IL-1Ra/IL-1β, were tested. The ratio does not change the model behaviour since the fraction of IL-1β bound receptors *F* rather than the level of IL-1β determines the inflammatory stimulus. However, the choice of the tissue-blood ratio affects the values of the model parameters (*λ and k*_1–6_) derived from steady state conditions.

**Table 2:** Steady state conditions for the IL-1β/IL-1 Ra subsystem.

	Glucose(mM)	IL-1β (pg/ml)	IL-1 Ra (pg/ml)
	(G)	(L)	(A)
compensation	5.47	12	6000
bifurcation	5.84	*L_b_* (unknown)	*A_b_* (unknown)
overt diabetes	13.4	12*55.8	6000*1.8

#### 2. Steady state of β-cell mass determines *λ*

β-cell proliferation and apoptosis are assumed to be exactly balanced at a healthy glucose level (4.5 mM), which determines the parameter *λ* assuming Eq. (12) in steady state using *F*_0_(G_0_ = 4.5 mM). If the glucose level is less than 4.5 mM, the apoptosis rate would surpass the proliferation rate which would decrease the β-cell mass. When the glucose level increases, more IL-1β is produced which stimulates β-cell proliferation and increases the β-cell mass. Increasing insulin resistance detunes this equilibrium.

#### 3. Steady state at the critical (bifurcation) point

A sudden rapid increase in fasting glucose happens about two years before diagnosis of T2D. The average fasting glucose level at the inflection point of glucose history is 5.84 mM [5] (Table 2).

#### 4. Steady state in the overt diabetes phase

IL-1β mRNA expression was measured in β-cells from T2D and non-diabetic cadaver donors [17]. Real-time quantitative PCR revealed in average 55.8 fold more IL-1β mRNA in T2D patients compared to healthy controls. As discussed above, we assume a linear relation of mRNA and protein concentration. The average fasting glucose of those diabetic donors was 13.4 mM (Table 2) [17]. Because of the bimodal dependency of IL-1Ra on IL-1β (see Fig. 2E in [32]), two IL-1Ra levels may be consistent with the 55.8 fold increase of IL-1β: Either the basal level of IL-1Ra or a 1.8 fold increased level. We tested both solutions by parameter scanning (see below) and only the latter solution yielded consistent results and is, therefore, taken in the model (Table 2).

#### 5. Steady state equations

The steady state equations for Eq. (2) and (3) read
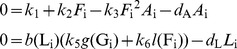
where i = {c,b,d} correspond to the compensation, bifurcation and diabetes state, respectively. The values are summarized in Table 2. Note that IL-1β (*L*_b_) and IL-1Ra (*A*_b_) are not determined by experimental data.

#### 6. Determine the range of IL-1β and IL-1Ra at bifurcation by parameter scan

**Figure 3:**
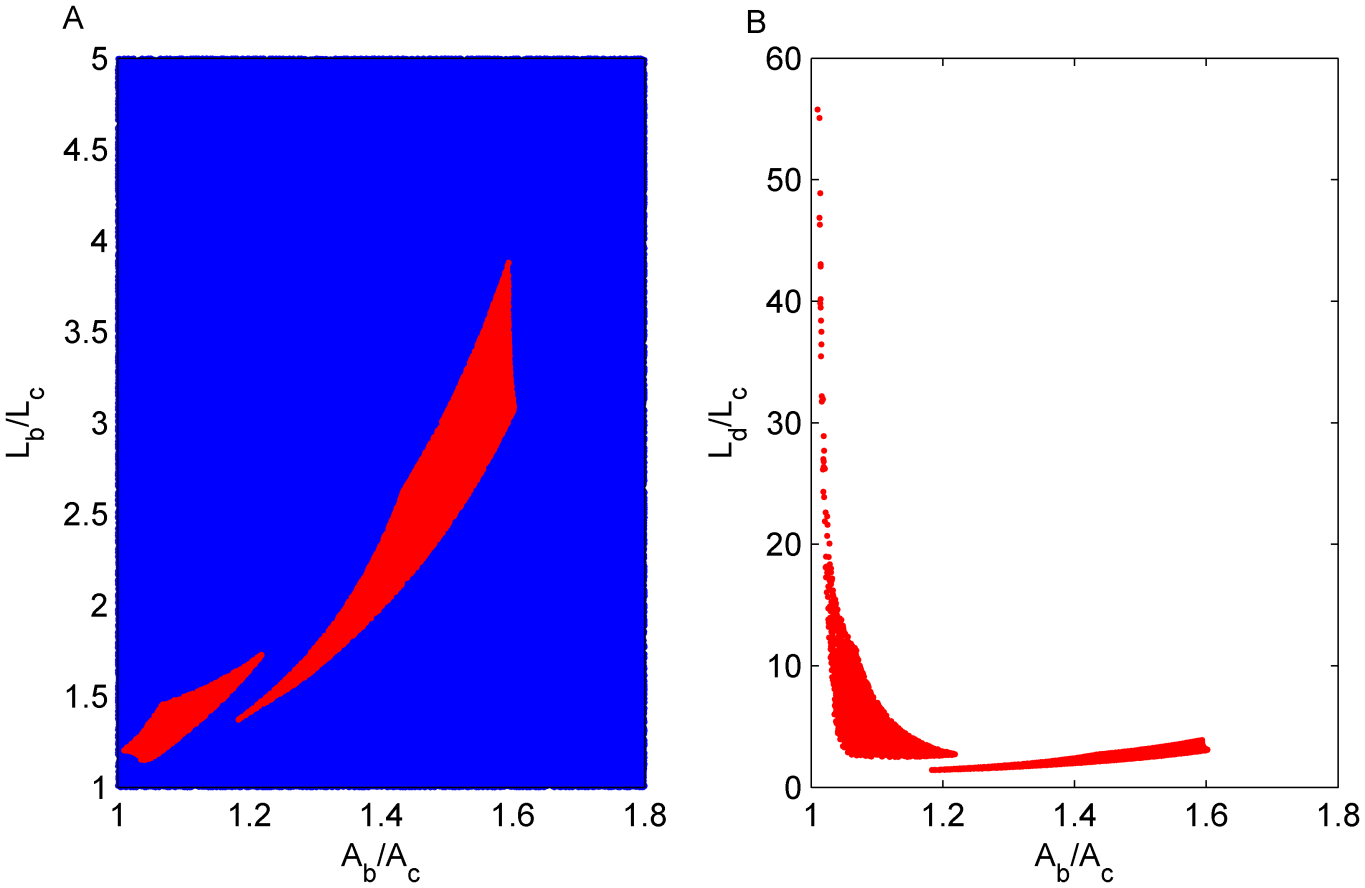
Parameter determined by scanning. A: Random points are selected in the *L*_b_-*A*_b_ plane (normalised with *L*_c_ and *A*_c_) and used in the steady state equations (Eq. (2,3)) to determine *k*_1–6_. If *k*_1–6_ are non-negative, points either induce an IL-1β switch (red) or do not (blue). B: The IL-1β level *L*_d_/*L*_c_ after the switch is shown as a function of IL-1Ra concentration at the bifurcation point.

The *L*_b_-*A*_b_ parameter plane is scanned in order to identify possible IL-1β and IL-1Ra concentrations at the bifurcation point. The values are restricted by the side condition that a switch of IL-1β and IL-1Ra exists (Fig. 3 A and B). This is done by numerically checking the determinant of the Jacobian and then checking the number of solutions on both sides of the bifurcation point *G_b_* (the Matlab script is included in Dataset S1). While a switch of IL-1β exists in a large area of the *L*_b_-*A*_b_ plane (red region, Fig. 3 A), the required switch to a 55.8 fold IL-1β level is only achieved for a small subset of values (Fig. 3 B). Most parameter combinations led to a smaller switch of IL-1β. This determined the choice of the values for *L*_b_ and *A*_b_. Note that the scan for different basal IL-1β/IL-1Ra levels (120/6×10^4^ or 1200/6×10^5^ with IL-1Ra/IL-1β ratio fixed at the reported value [27]) yielded similar results (Fig. 3).

**Figure 4:**
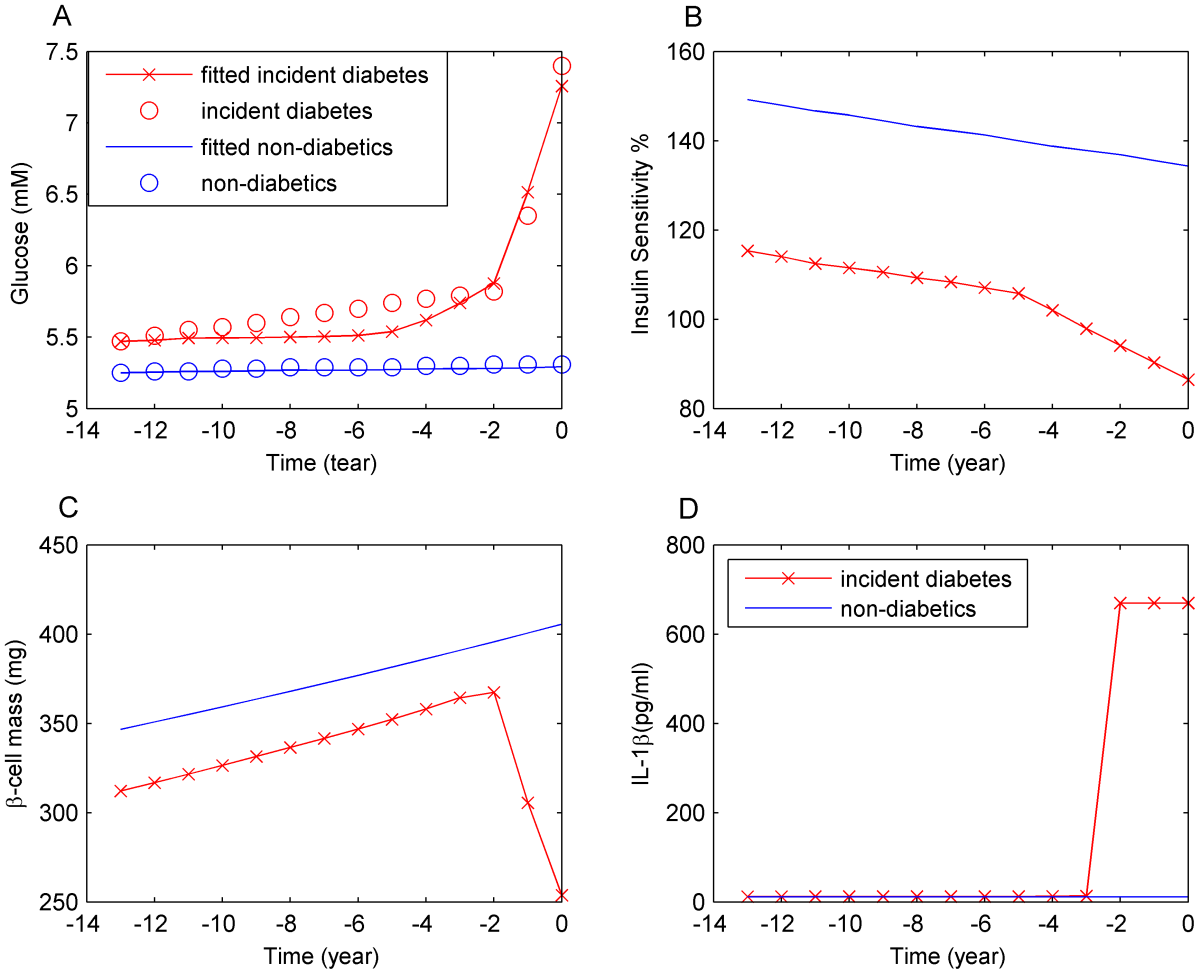
Fitting the model to the fasting glucose history to determine *m* and *τ*. Shown are the best fit results of incident diabetes cases before T2D diagnosis and the data (black line) (red line cross) and non-diabetic controls (red line) (A), interpolated insulin resistance as model input (B), corresponding behaviour of β-cell mass (C) and IL-1β (D).

### Fitting the natural history of fasting glucose

**Figure 5:**
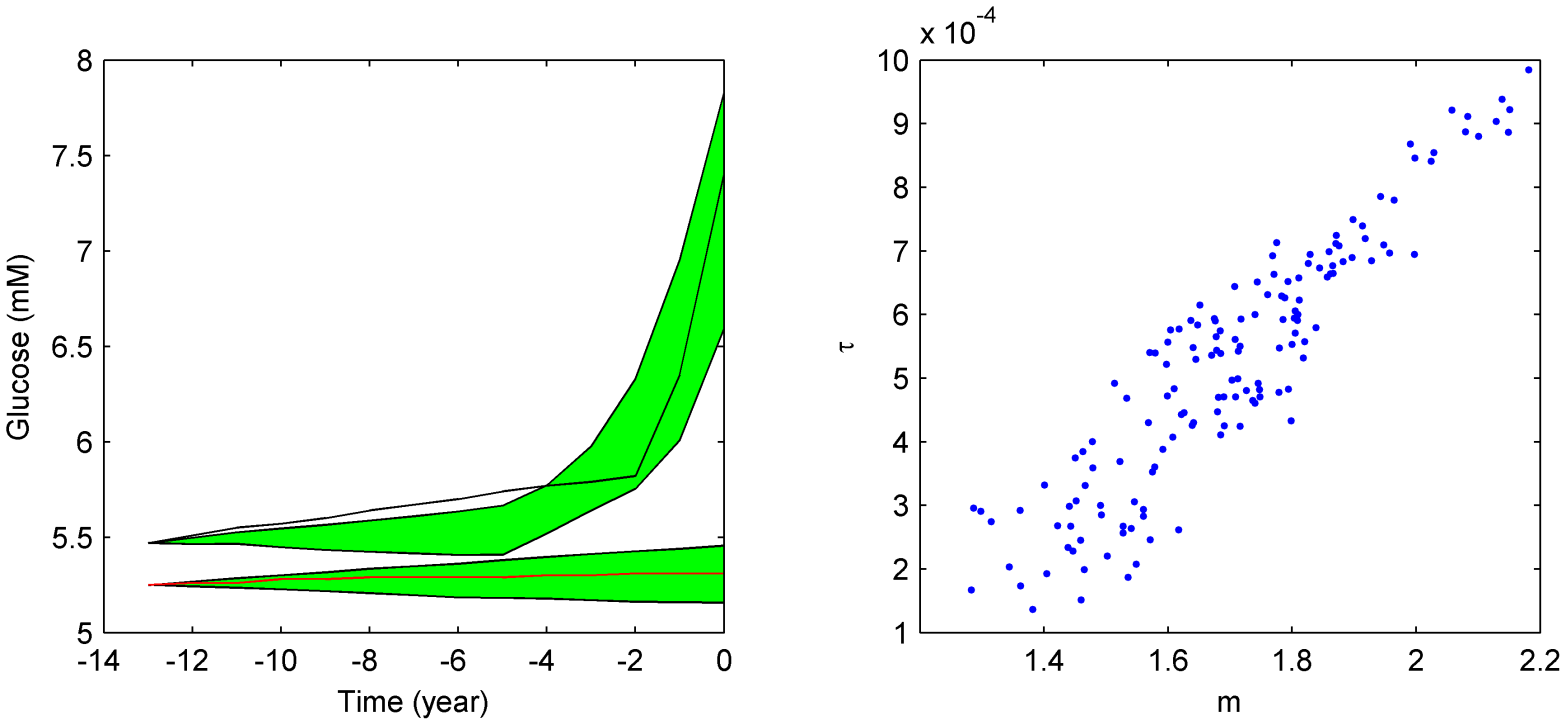
There exist multiple reasonable fits (shadow) of incident diabetes cases before T2D diagnosis and the data (black line) and non-diabetic controls (red line) (A). Fitted parameters exhibit correlations (B).

The last two undetermined parameters, *m* and *τ* defined in Eq. 11 and 12, are determined by fitting the model to the fasting glucose history of both incident diabetes cases and the non-diabetics controls [5]. The best fit is shown in Fig. 4A. Interpolated insulin sensitivity [5] (Fig. 4B) which is an input to the model during fitting, the behaviour of the β-cell mass (Fig. 4C) and of IL-1β (Fig. 4D) are also shown. The fitting is implemented in the SBTOOLBOX2 [40] using a differential evolution algorithm, which reports multiple possible parameter sets (Fig. 5A). A strong correlation between *m* and *τ* was identified (Fig. 5B), which gives rise to large variations of glucose and β-cell mass in the simulations of overt diabetes (Fig. S1). In other words, the pre-diabetes glucose trajectory does not allow for a precise determination of *m* and *τ*. The values from the best fit are reported in Table 1 and used throughout the paper.

### A complementary approach

The above approach demonstrates that the bifurcation of the IL-1β/IL-1Ra subsystem is sufficient to account for the fitted data set. To investigate whether the bifurcation of the IL-1β/IL-1Ra subsystem is necessary, a complementary approach is employed: the model is fitted to the data without bifurcation as a presupposed condition. The steady state equations for Eq. (2) and (3), in the compensation phase and in the overt diabetes phase, allow reducing the degree of freedom from 8 (*k_1∼6_*, *m* and *τ*) to 4: *k_1_*, *k_2_*, *k_5_*, and *k_6_* are explicitly expressed as functions of *k_3_* and *k_4_*, and only *k_3_*, *k_4_*, *m* and *τ* are fitted. Strong correlations are found to exist between *m* and *τ*, which cause large variations of glucose and β-cell mass in the simulations of the overt diabetes (Fig. S2 A and B). Strong correlations are also found between *m* and *k_4_*, which cause large variations of the time point of the IL-1β/IL-1Ra switch (Fig. S2 C and D). Please note that the complementary approach found, although with low probability, cases allowing an extra transition (see Fig. S2 C and D).

Although some parameters cannot be determined precisely, the key qualitative property, i.e. the switch of the IL-1β/IL-1Ra subsystem before T2D diagnosis, remains robust. Taking together the results of both complementary approaches, it is suggested that the bifurcation of the IL-1β/IL-1Ra subsystem is both sufficient and necessary to account for the data set, in the framework of the current model. Please note that despite the model being validated by quantitative experimental data, the results presented here remain of qualitative nature. In particular, in the overt T2D state we expect that additional physiological process as well as medication will alter the results on a quantitative level.

## Results

### The IL-1*β*/IL-1Ra subsystem has multiple steady states

**Figure 6:**
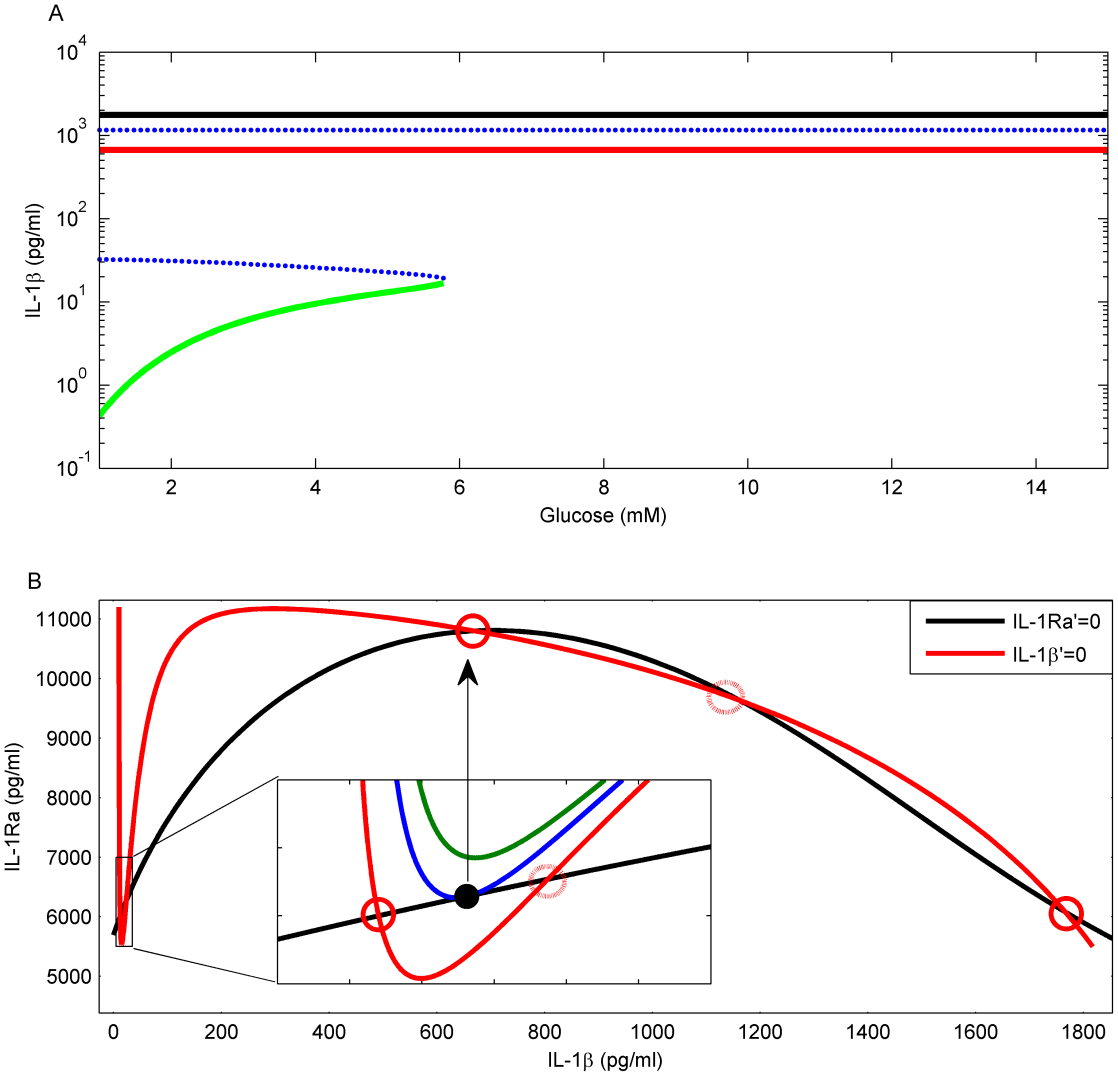
A: The bifurcation diagram of the IL-1β-IL-1Ra subsystem as glucose (*G*) varies. This subsystem exhibits three stable (full lines) and two unstable (dotted blue lines) steady states. Above a critical glucose level (5.84 mM) the system loses stability of the pre-diabetic state (green line) and progresses to a high IL-1β state (red line), which is stable across the whole physiological glucose range. A third stable state (black line) with even higher IL-1β exists, which, in the hyperglycaemia range, may be associated with advanced T2D. B: A transition from five to three steady states is found that corresponds to the transition from pre-diabetes to overt T2D. Steady states appear as crossing points of the nullclines. The nullclines of IL-1β (red) and IL-1Ra (black) cross in five points at normal glucose level (4.5 mM). When glucose increases, the IL-1β nullcline raises its minimum (inset, transition from red to blue to green curve) such that the low IL-1β and low IL-1Ra steady state vanishes. The system is forced to switch to a high IL-1β state which may be associated with overt T2D. The corresponding glucose level is 4.5, 5.84 and 6.84 mM for the red, blue and green IL-1β nullcline in the inset, respectively.

An IL-1β switch is observed in a large range of parameter values including the physiological relevant range. Below the critical glucose level of 5.84 mM the model gives rise to five steady states, three stable and two unstable ones (Fig. 6A). The stable steady state with lowest IL-1β (green in Fig. 6 A) is stable only if glucose is below the critical level. It is interpreted as corresponding to the compensation phase. The hyperglycaemia parts of the two other stable steady states may be associated with overt T2D.

Nullcline analysis facilitates understanding the transition between the different inflammatory levels. Steady states appear as the crossing points of the IL-1Ra versus IL-1β nullclines where both quantities become static (Fig. 6B). The IL-1β nullcline (Fig. 6B red line) exhibits a nadir followed by a peak, which is the typical feature of an auto-stimulation species. The IL-1Ra nullcline (Fig. 6B black line) exhibits one peak. This implies that at large IL-1β its further increase is accompanied by decreased IL-1Ra. The measured non-linear dependence of IL-1Ra on IL-1β [32] is at the origin of the third stable steady state with highest IL-1β. If IL-1Ra is stimulated by IL-1β in a linear manner, only two stable steady states would have been found. Such a dysregulation of IL-1Ra may also happen in other diseases involving IL-1β, such as chronic myelogenous leukaemia and hairy cell leukaemia [29]. Hence, dysregulation of IL-1Ra at high IL-1β concentrations might be associated with a boost of disease progression.

### The compensation steady state is lost upon progression to overt T2D

The stable steady state with lowest IL-1β corresponds to the pre-diabetic state. As glucose influences IL-1β production [14], [17] the nadir of the IL-1β nullcline moves up in response to increased glucose (Fig. 6B inset) while the IL-1Ra nullcline remains unchanged (Fig. 6B, black line). The two involved steady states approach each other and ultimately merge into one steady state (Fig. 6B inset, blue line). At even higher glucose this steady state disappears and the system makes a transition (Fig. 6B, black arrow) to the next available stable steady state, which is characterised by substantially higher IL-1β and moderately increased IL-1Ra. This transition is interpreted to correspond to the sudden transition from the compensation phase to disease progression.

At even higher levels of IL-1β, the effect of glucose on IL-1β production is inhibited by IL-1β itself (represented in Eq. 3 by *b*(l)), such that the IL-1β nullcline remains unaffected by glucose at high IL-1β concentrations. Therefore, the current model only reflects the transition from the compensation phase to the mild disease phase and not to the strong disease phase. An extended model including detailed IL-1β mRNA dynamics and other factors like free fatty acids, leptin or tissue resident macrophages might generate two transitions.

### Glucose-lowering therapy cannot cure or stop progression of T2D

**Figure 7:**
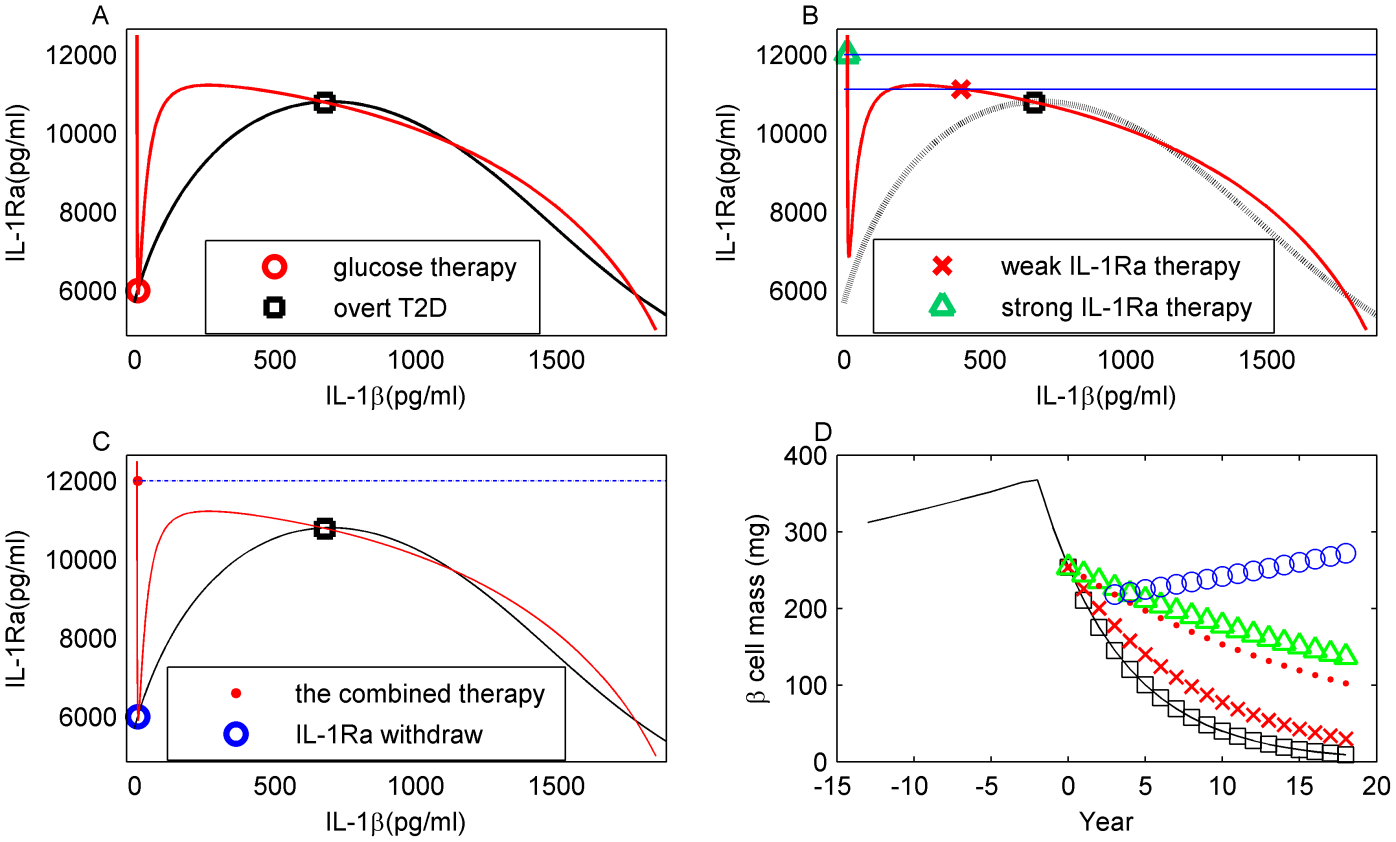
T2D therapy strategies and their effect on β-cell mass. A: Glucose-lowering therapy. The pre-diabetic steady state is restored at the IL-1β (red line) and IL-1Ra (black line) nullcline intersection (red circle). But the system stays in the stable T2D steady state (black square). B: Permanent IL-1Ra therapy. The IL-1β (red line) and IL-1Ra (black dotted line) nullclines do not intersect at low IL-1β and the system starts in the stable overt T2D steady state (black square). Clamping IL-1Ra to levels below the peak of the IL-1β nullcline (lower blue line) reduces IL-1β (red cross). Clamping IL-1Ra to levels above the peak (upper blue line) makes IL-1β jump to low levels (green triangle). C: Combined IL-1Ra and glucose-lowering therapy. Above threshold clamp of IL-1Ra (dashed dotted line) induces a substantial IL-1β reduction (red dot). Simultaneous control of glucose restores a stable steady state at low IL-1β (red and black nullclines intersect at the blue circle). A release of IL-1Ra clamp induces a transition to this steady state. D: Effect of the different treatments on β-cell mass. Therapy as in A (black squares), B (red crosses for below threshold IL-1Ra clamp; green triangles for above threshold IL-1Ra clamp), C (red dots for combined IL-1Ra and glucose-lowering therapy; blue circles after release of IL-1Ra clamp). No treatment (black line).

Glucose-lowering therapy, simulated *in silico* by fixing the level of glucose at normal level (5.47 mM), is represented by the IL-1β nullcline which now, as in the pre-diabetic state, intersects with the IL-1Ra nullcline (Fig. 7A) around its nadir at low IL-1β. The therapy reconstitutes the existence of the pre-diabetic stable steady state (Fig. 7A, red circle). However, the original pathological steady state (Fig. 7A, black square) is neither deleted nor does it lose stability. The disease state remains stable irrespective of the glucose levels reached by glucose therapy. Therefore, the glucose-lowering therapy does not induce a transition from overt T2D to pre-diabetes and the functional β-cell mass is further decreasing (Fig. 7D, black squares).

### The pre-diabetic state cannot be restored by IL-1Ra therapy of overt T2D

IL-1Ra is a naturally occurring inhibitor of IL-1β, and it is upregulated in obesity [28] and reduced in poorly controlled T2D patients [27]. By injecting exogenous IL-1Ra, IL-1Ra therapy aims at inhibiting IL-1β signalling. This is simulated *in silico* by fixing the level of IL-1Ra at a higher concentration (Fig. 7B, red cross). The simulated therapy slows down the loss of β-cell mass (Fig. 7D, red crosses) because increased IL-1Ra drives IL-1β into a less pathological regime. The higher IL-1Ra is set, the more IL-1β is reduced. At a threshold level of IL-1Ra, which is determined by the peak of the IL-1β nullcline (Fig. 6B, red line), the response of IL-1β becomes qualitatively different and it is reset below its physiological normal level (Fig. 7B, green triangle). Though the speed of β-cell loss is significantly reduced, it is not stopped (Fig. 7D, green triangles). Releasing IL-1Ra fixation leads to a restoration of the T2D state associated with accelerated loss of β-cell mass (Fig. 7B, black square), because the stable pre-diabetic steady state associated with low IL-1β is not restored by this therapy (Fig. 7B).

### The pre-diabetic state can be restored by combined glucose-lowering and IL-1Ra therapy

A combined IL-1Ra and glucose-lowering therapy (Fig. 7C) is required to induce functional β-cell mass reconstitution. Strong IL-1Ra therapy induces a transition from the overt T2D steady state to a transient state at low IL-1β (Fig. 7C, from black square to red dot) associated with reduced speed of β-cell loss (Fig. 7D, red dots). A concurrent glucose-lowering therapy restores a stable steady state at low IL-1β (Fig. 7C, blue circle). Upon withdrawal of the IL-1Ra fixation, the *in silico* patient makes a transition to the restored stable steady state (Fig. 7C, from red dot to blue circle) which is associated with β-cell growth (Fig. 7D, blue circles), thus, inducing a long-standing improvement of the disease state.

### The IL-1Ra therapy must be released to achieve a disease state improvement

Long-term combined IL-1Ra and glucose-lowering therapy prohibits β-cell mass regrowth *in silico* (Fig. 7D, red dots). The model predicts that IL-1Ra therapy, simulated in the model as the fixation of IL-1Ra, must be released in order to allow for functional β-cell mass reconstitution. β-cell mass regrowth is suppressed during IL-1Ra therapy as it reduces IL-1β to levels below the pre-diabetic state.

## Discussion

An IL-1β switch model of T2D onset after long-term compensation was presented and a strategy for an anti-inflammatory therapy of T2D was proposed. The model is characterised by a pre-diabetic state during which increasing insulin resistance drives hyperglycaemia. This ultimately leads to a changed steady state configuration and progression into the overt T2D disease state. The model parameters are derived from *in vitro* islet measurements, from steady state conditions, and from glucose history of T2D patients, such that the model parameters are fully determined. In the following we first discuss its relevance to the empirical theory of five-stage β-cell dysfunction [3], then describe the predictions of the model, its relevance and potential contributions to clinical trials [23], [26], and its relation to previous modelling works [33], [35]. Last but not least, we discuss its limitations.

Interestingly, the model shows three steady-states of IL-1β. This result is beyond our expectations when designing the model but is consistent with the five-stage diabetes development theory [3]. This theory is mainly based on clinical observations, where stage 2, “adaptation” (termed as compensation in this paper) corresponds to the model state of lowest IL-1β (green in Fig. 6A). Stage 3 “transient unstable early de-compensation” corresponds to the sudden switch of IL-1β/IL-1Ra in the model, where a 55 fold increase in IL-1β level is accompanied with only 2 fold more IL-1Ra, and the transient adaptation time thereafter. Stage 4 “stable de-compensation” corresponds to the state with medium IL-1β level (red in Fig. 6A) and stage 5 “severe de-compensation” corresponds to the state with highest IL-1β level (black in Fig. 6A). Stage 1 “normal” is not represented in the model because glucose level is already higher even 14 years before T2D diagnosis (see Fig. 4A and [5]). Thus, the IL-1β switch model qualitatively reflects all stages of T2D development, starting from the compensation phase and progressing to the mild disease and ultimately to the strong disease state.

Stage 3 is observed to be transient, persisting a few weeks in different animal models that were subjected to partial pancreatectomies [41] or islets transplantation [42], [43]. Interestingly, islets transplantation [43] and pancreatectomies [41] in rodents gave rise to glucose levels in two discrete groups: either normal glucose or severe diabetes, with almost no glucose levels in between, a pattern consistent with the idea of two distinct states in the presented model. Stage 3, transient and unstable, lies between the clinically non-pathologic and pathologic states, and thus, may be of great interest in terms of providing a time window during which therapies are more efficient in reversing the pathological processes right after diagnosis. Results from newly diagnosed diabetic patients who received intensive insulin therapy supports this notion [31]. However, in the current model, the switch of IL-1β/IL-1Ra, which is relevant to stage 3, is instantaneous (see Fig. 6 and Fig. S2) because the model does not describe the dynamics of the IL-1β/IL-1Ra subsystem during the transition. The equations of the IL-1β/IL-1Ra are mainly based on assumed steady state relations, which were derived from in vitro data. In addition, the model does not describe the dynamics of insulin resistance, the improvement of which has been assumed to be responsible of preventing/postponing the transition during stage 3 [3]. Rather, insulin resistance is implemented as an input to the glucose/insulin subsystem. These limitations of the current modeling approach (see also later discussions about the meaning of *G* and *I* in the model) make information about the duration of stage 3 unavailable from the model.

The IL-1β bi-stable switch model suggests four requirements for a successful anti-inflammatory treatment of T2D:

1. Glucose-lowering therapy is combined with IL-1Ra therapy;
2. IL-1Ra is kept at an above threshold level;
3. IL-1Ra fixation is released when IL-1β levels are down;
4. Glucose-lowering therapy is continued on long-term.

Then, the functional β-cell mass is predicted to increase. But even if it reaches its healthy level, the *in silico* T2D patient would remain in the pre-diabetic state, since the release of glucose control would restart T2D progression due to the pathological state of insulin resistance. The power of this therapy is not to cure T2D but to keep the *in silico* patient in the pre-diabetic state.

Dose and duration of anti-IL-1β therapy are critical. On the one hand, an above threshold IL-1Ra is required for inhibition of IL-1β auto-stimulation and the reverse transition from β-cell failure to compensation. On the other hand, IL-1β has to be kept above its healthy level for β-cell reconstitution. This infers a U-shaped dose-effect relationship of anti-IL-1β therapy *in silico*, which has been confirmed in a phase 1 clinical trial using a monoclonal antibody of human IL-1β [23].

**Figure 8:**
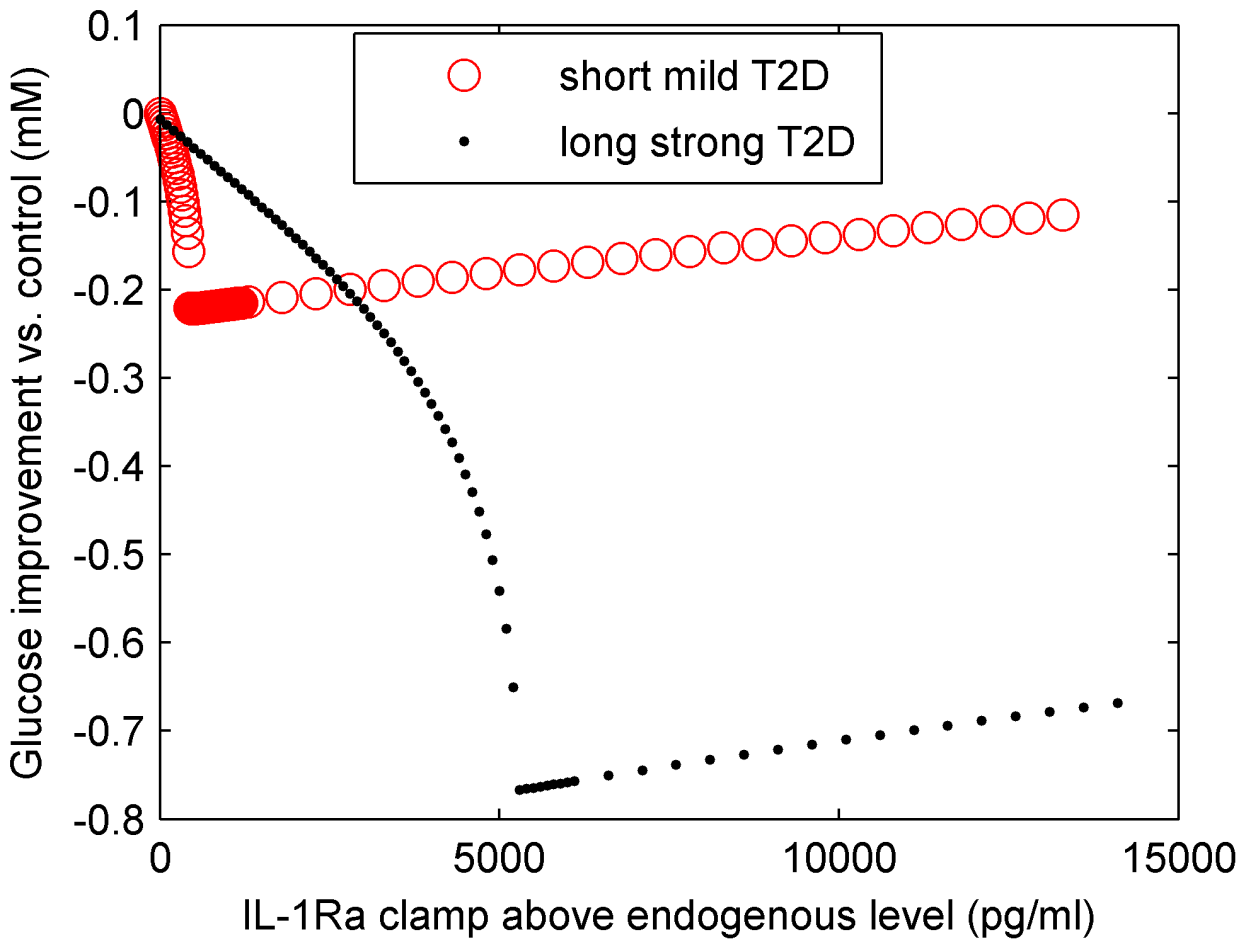
The dose-effect relationship of IL-1Ra therapy for mild (red circle) and strong T2D (black dot) *in silico* are shown. Following the protocol of the clinical trial, glycaemia improvement after 90 days of therapy versus control, is used as a measure. The IL-1Ra threshold predicted by the model is reflected in the jump of the resulting glucose level for both T2D groups. The optimal IL1-Ra dose for strong T2D is substantially larger because of the lower endogenous level of IL-1Ra. The best achieved glucose improvement in response to the therapy is three-fold higher in the case of strong than in mild T2D. The starting glucose level is 11.96 and 18.02 mM for mild and strong T2D, respectively. The final glucose level of the both control groups is 12.21 and 18.82 mM, respectively.

Large scale clinical trials with anti-IL-1β agents revealed that glycaemia was less efficiently reduced in patient groups with short (6 years) compared to groups with long (11 years) disease duration [26]. We have performed simulations of IL-1Ra therapy on patients with short and long T2D duration, where short and long T2D are associated with mild and strong T2D steady states, respectively (Fig. 8). The strong T2D state is initiated by manually increasing IL-1β and decreasing IL-1Ra to the levels shown in Fig. 6B at 11 years. Then, anti-IL-1β therapy (ignoring other adjuvant medication) is applied by an IL-1Ra fixation for 90 days to both *in silico* patient groups. The IL-1Ra dose-effect relationship for the two groups, expressed by the glycaemia improvement versus control, is compared (Fig. 8). In agreement with the clinical-trials results, optimal IL-1Ra dosage induced more efficient glucose improvement for strong than for mild T2D. This happens because in strong T2D the aggravation of blood glucose levels is faster in the control group. The optimal dose of IL-1Ra is different in both groups *in silico* (Fig. 8). This calls for an individualised treatment according to the endogenous inflammatory state. These simulations do not aim at explaining the diverse outcomes from clinical trials because other adjuvant medications are neglected during the simulations. Furthermore, the capability of the current model is limited to the low to medium hyperglycaemia/IL-1β region (see later discussion). However, the model still provides a framework in which different outcomes can be discussed in respect to patients' internal inflammatory levels and strategies may be suggested for patient stratification with respect to their inflammatory levels at the beginning of a clinical trial.

The model proposed by De Gaetano et al [35] is able to account for different arms of the Diabetes Prevention Program (DPP) results [44], by modeling the effects of different drugs on insulin resistance development. Some differences and similarities are worth noting here, besides those already discussed in the introduction. In both models glucose toxicity introduces a positive feedback in the disease progression. Therefore, both models emphasize the importance of an early treatment of hyperglycaemia. The unique property of the current model is that the inflammatory signal, once switched on by glucose, cannot be switched off by glucose-lowering therapies. It has to be treated separately. This point seems to be consistent with recently discovered anti-inflammatory effects of some long-standing anti-diabetic agents. In fact, insulin has long been known for its anti-inflammatory effect, which, early presumed to be due to its glucose-lowering ability, recently was established to be a complement to its metabolic role [for a recent review see 45]. Additionally, there is emerging evidence suggesting that metformin, the first-line diabetic drug, have anti-inflammatory effects that are independent of its hypoglycaemia effect [46], even though its anti-inflammatory effect in T2D being not as robust as in other diseases, such as hypertension [47]–[49]. The impact of currently employed T2D drugs onto the inflammatory state of the patients prohibits falsification or verification of the model prediction that the T2D disease state cannot be alleviated by controlling glycaemia alone.

The presence of the IL-1β switch suggests that the system is operating in two distinct regimes, which defines a clear threshold for an anti-inflammatory therapy. A striking prediction of the model is that the anti-inflammatory therapy has to be released after a certain time. These inferences are derived from the IL-1β/IL-1Ra hysteresis and are consistent with some long-standing findings [11], [12], as well as recent progresses in the field, such as multistage development of the disease [3], [5], long-term effect of temporary IL-1Ra in overt-diabetes [20] and the efficiency of glucose control in pre-diabetes but not overt-diabetes [31], [36].

The present IL-1β switch model is built on *in vitro* data and several assumptions, both of which cast limitations on its applicability. The *in vitro* data show regulation of IL-1β and IL-1Ra by glucose and exogenously added IL-1β [14], [17], [27], [32]. Other factors involved in *in vivo* IL-1β control, such as free fatty acids [50], infiltrated macrophages and islet amyloid polypeptides [51] are not considered. These factors are associated with serious hyperglycaemia and overt T2D. However, they do not or only weakly affect the results of the core IL-1β model, especially concerning the onset bifurcation at glucose levels below 6 mM.

IL-1β induced β-cell proliferation and apoptosis were extrapolated from data of cultured islets [32]. It is unclear to what degree the *in vitro* data can reflect relevant *in vivo* processes, but several weak points of this assumption could be delineated. There is evidence that β-cells can re-enter cell cycle *in vivo* and that β-cell mass can adapt to metabolic needs [52], both supporting adaptive β-cell proliferation *in vivo*. However, it is not the only possible source of functional β-cells. Dedifferentiation of other cell types with a common precursor [53], as well as reactivation of dysfunctional β-cells [8], contributes to β-cell homeostasis. Furthermore, data from autopsy indicate that the proliferation rate of existing β-cells is very low. Consequently, increased β-cell mass seen in the compensation phase may be mainly attributed to new islet formation from other cell types [54]. A more realistic description of β-cell mass dynamics may further improve the model and extend its validity to later stages of the disease.

By representing the inflammatory stimulus via *F* instead of IL-1β, it is implicitly assumed that the amount of membrane IL-1β receptor per β-cell is constant. In support of this, there is evidence that the amount of IL-1β receptor in the β-cell membrane is much higher than in other cell types [55], suggesting that it is saturated. However, it is unlikely that the receptor is not down-regulated at high concentrations of IL-1β. Consequently, the model may overestimate the inflammatory signal at high IL-1β levels. This limitation has to be kept in mind when interpreting model results at high IL-1β.

Another important assumption concerns the IL-1β mRNA quantity used in the model. The IL-1β mRNA quantity is assumed to be proportional to the IL-1β protein level, in order to establish the quantitative relationships in IL-1β production. However, IL-1β production is characterized by the dissociation of transcription and translation. As a result, high levels of IL-1β mRNA are always associated with low levels of protein [29]. Alternatively, a sigmoidal relationship may be considered instead of the linear one used here. While it is difficult to estimate the quantitative error induced by the assumed linear relationship, it is straightforward to predict that the alternative sigmoidal relationship would increase the nonlinearity of IL-1β auto-stimulation, and of the interaction between glucose and IL-1β. Therefore, the essential elements for the bi-stable switch remain unchanged (or even would be increased), such that the qualitative results of the IL-1β switch model are robust.

It is difficult to define the exact physiological meaning of *G* and *I* in the model [35]. Since *G* and *I* are described by one-compartment dynamics in the model, only changes in long-term fasting levels are accessible to the model. Hence, we propose to interpret *G* as an abstract parameter associated with long-term fasting glucose on a time scale of days to weeks. Fasting levels represent more the internal property of the body's glucose control system than the history of how it was perturbed by meal or exercise [56]. However, the driving stimulus of IL-1β production may depend on peaks of glycaemia rather than on fasting levels. It is possible that dysregulations of fasting glucose levels and postprandial peaks are independent, as evidenced by patients with impaired glucose tolerance but normal fasting glucose. Eventually, the modifications of the dynamics of insulin release (first-phase insulin) are more important for glycaemia control than the long-term fasting levels of glucose. We would like to emphasize that these details of glucose and insulin dynamics are beyond the resolution of the model presented here and have to be addressed with a model focusing on the short-term dynamics of these quantities.

T2D is a very complex disease. The current model is simplified on purpose in order to generate structural insight. Other players of the glucose control system, for example glucagon, also play very important roles in glucose homeostasis and T2D development. Augmented glucagon secretion, together with impaired β-cell function, happens in the very early phase of pre-diabetes [57], [58]. The underlying islet defects were identified as reduced maximal insulin response and reduced glucose-sensitivity of β-/α-cell [58]. According to the physiological integral rein control theory [56], [59], glucose-sensitivity of β-/α-cell are important factors in setting steady state glucose level. In fact, recent studies support an independent role of glucose-sensitivity in hyperglycaemia development [60], [61]. Future improvement of the model should include the glucose-sensitivity of β-/α-cell to account for the rising of fasting glucose in the compensation phase. In particular, this might improve the fitting to the data in Fig. 4A during the compensation phase.

In addition, post-load glucose of incident diabetes cases showed a rapid increase 5 years before diagnosis [5], which is followed by a rapid decrease in insulin sensitivity. This important change is not reflected in the history of fasting glucose while it is represented in the model output (Fig. 4A). On the one hand, the absence of a corresponding change in fasting glucose at ∼5 years before diagnosis suggests that fasting glucose level is dominated by factors other than insulin resistance. Again, this might point to a role of glucagon. On the other hand, the coincident post-load glucose increase and insulin sensitivity decrease at ∼5 years before diagnosis might suggest a cause-effect relationship between β-cell function and insulin resistance [62]. The complexity of the defects during the pre-diabetes phase highlights the necessity of the mathematical modeling approach in the field.

Although results from both in vitro experiments and animal models were promising, the actual effect of anti-IL-1β agents in clinical trials of T2D was a matter of debate because the observed effect was very modest so far. Besides the possible role of polymorphisms of the IL1RN gene which encodes IL-1Ra [20], more research is needed to clarify for example, drug interaction with other IL-1 family members, such as soluble IL-1 receptors and soluble IL-1 receptor accessory proteins, in the pancreatic local environment as well as in blood and liver.

On the structural level, the present analysis proposes that the transition from pre-diabetes to overt T2D is associated with changed stability properties of the endocrine-immune system. The transition is initiated at a threshold value of glucose and associated with qualitatively different inflammatory states. The notion of an IL-1β hysteresis is supported by data from different studies, which showed the effectiveness of glucose control in pre-diabetes [36] or newly diagnosed patients [31] and the ***in***effectiveness of glucose control in long-standing T2D patients [11]–[13]. It is also supported by the clinical trial based on a combined IL-1Ra and glucose-lowering therapy: the improvement of the disease state remained for a year after release of the IL-1Ra therapy [20]. The existence of the IL-1β hysteresis requires a combined glucose-lowering and anti-inflammatory therapy for overt T2D patients, where the anti-inflammatory therapy should be kept for only a limited time.

## Supporting Information

Dataset S1 This zip files contains the matlab scripts for reproducing the figures in the manuscript. It contains a readme.txt where more details are present.
(ZIP)

Figure S1 Simulation of glucose (A) and β-cell mass (B) evolution to overt-diabetes based on multiple fitted parameters. Bifurcation is used as a side condition.
(EPS)

Figure S2 Simulation of glucose (A), β-cell mass (B), IL-1β (C) and IL-1Ra (D) evolution to overt-diabetes based on multiple fitted parameters. Bifurcation is not used as a side condition.
(EPS)
